# Giant Lipoma Presents from the Buccal Vestibule

**DOI:** 10.1155/2020/8824548

**Published:** 2020-10-30

**Authors:** Yuki Sakamoto, Gohei Oyama

**Affiliations:** Departments of Oral Surgery, Hospital of Hironokogen, 6512215, Japan

## Abstract

A lipoma is a benign tumor, where the parenchyma is composed of adipose tissue. Lipomas comprise 0.1%–5% of all benign tumors in the oral cavity. A 43-year-old man, with a known masticatory disorder, visited the Department of Oral Surgery at our hospital because of a facial swelling. The swelling, which had worsened over 15 years, was not painful, but the patient had discomfort while eating and talking. A detailed examination revealed a smooth, elastic, soft, circular, and yellowish pink pedunculated tumor-like lesion, with a diameter of approximately 40 mm, on the right buccal mucosa. An oval-shaped neoplastic lesion with a well-defined border of 40 mm × 20 mm was noted on MRI. Based on these results, the lesion was characterized as being a benign lipoma. The tumor was resected under local anesthesia. The pedunculated tumor was excised with an electric knife, and the wound was sutured. No trismus or paresthesia was noted on the postoperative follow-up.

## 1. Introduction

A lipoma is a benign tumor, where the parenchyma is composed of adipose tissue. Lipomas often occur subcutaneously and present as tender, yellow masses under the submucosa. They commonly occur on the back, neck, thighs, and face, specifically the oral region. Lipomas comprise 0.1%–5% of all benign tumors in the oral cavity [1]. The site of development is the buccal mucosa, tongue, or floor of the mouth [2]. The most common site of lipoma is buccal mucosa. Lipoma growth is generally slow, thereby leading to delay in follow-up by the patient thus delaying diagnosis and treatment. Magnetic resonance imaging (MRI) allows for accurate diagnosis. The first line of treatment is resection. This is a case of a patient with a masticatory disorder, who presented with a large pedunculated lipoma, which was diagnosed 15 years after the initial presentation.

## 2. Case Presentation

**Figure 1:**
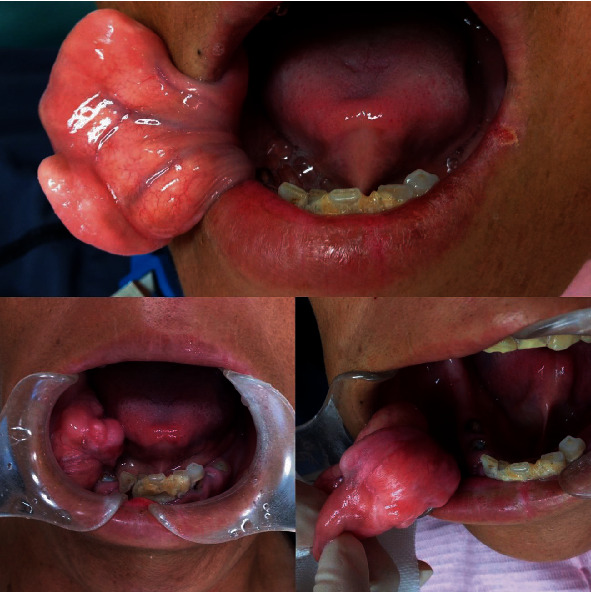
Intraoral photograph of the tumor. A pinky smooth, elastic, soft, circular, and pedunculated tumor-like lesion, with a diameter of approximately 40 mm, on the right buccal mucosa.

**Figure 2:**
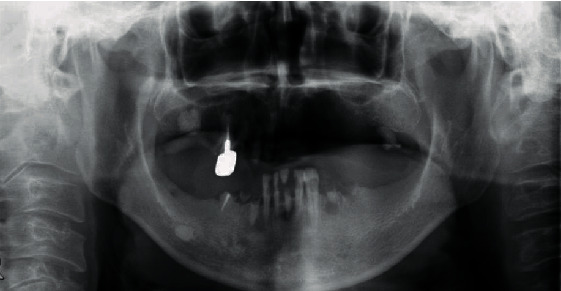
Panoramic X-ray photo. There are few teeth in his mouth, and he has difficulty eating.

**Figure 3:**
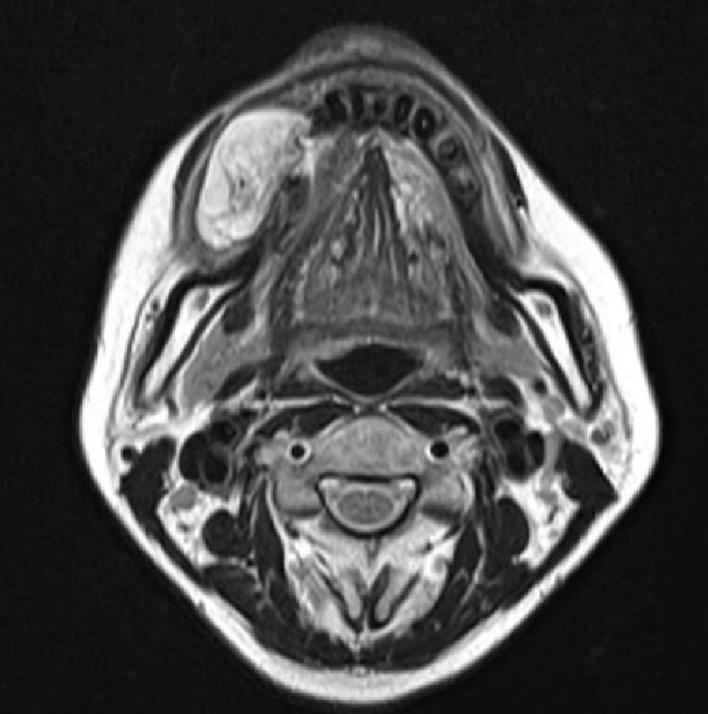
T2-weighted magnetic resonance image. The tumor shows high signal intensity on the T2-weighted image and an oval-shaped neoplastic lesion with a well-defined border of 40 mm × 20 mm.

**Figure 4:**
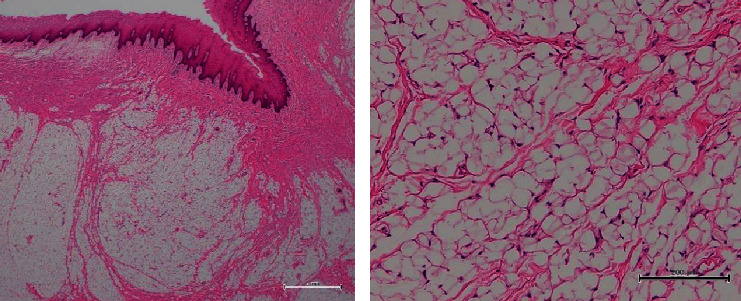
Histological examination. Connective tissue is found under the epidermis, and adipose tissue is filled underneath. Mature adipose tissue hyperplasia with blood vessels is noted in high magnification.

A 43-year-old man, with a known masticatory disorder in the oral phase and speech disorder, presented to the Department of Oral Surgery at Hironokogen Hospital due to facial swelling. The swelling, which had worsened over the course of 15 years, was not painful. The patient however experienced discomfort while eating and talking. A detailed examination revealed the presence of a smooth, elastic, soft, circular, and pedunculated tumor-like lesion, with a diameter of approximately 40 mm, on the right buccal mucosa. This resulted in swelling of the right cheek without any facial nerve palsy (Figure 1). We considered the following differential diseases by visual inspection, neurogenic tumor, liposarcoma, vascular tumor, and fibrous tumor. We took a panoramic X-ray photo, there were few teeth, and occlusion was collapsed. A light shadow that appeared to be a tumor is found under the residual root of the right maxilla. The panoramic photo did not show jaw bone resorption (Figure 2). An oval-shaped neoplastic lesion with a well-defined border of 40 mm × 20 mm was noted on MRI. The lesion was located between the epithelium and smooth muscle of the buccal mucosa. Both T1- and T2-weighted images showed high signal intensity (Figure 3). Fat suppression and diffusion-weighted images showed low signal intensity. The lesion was surrounded by tissue with low signal intensity on the T2-weighted image. The tumor is growing and pushing the buccal muscle and fascia without destroying normal surrounding structures. A malignant tumor is negative from the findings of MRI, and for the long course, it was determined that the lesion was a benign lipoma. The tumor was resected under local anesthesia, and a biopsy was submitted for microscopic examination. The pedunculated tumor was excised with an electric knife, and the wound was sutured. No trismus or paresthesia was noted on the postoperative follow-up. On histopathological examination, mature hyperplastic adipose tissue with blood vessels was noted (Figure 4). Further analysis revealed hyperplastic connective tissue hyperplasia as well. The tumor was classified as a lipoma. This study was granted an exemption from requiring ethics approval. Informed consent was obtained from the patient.

## 3. Discussion

**Figure 5:**
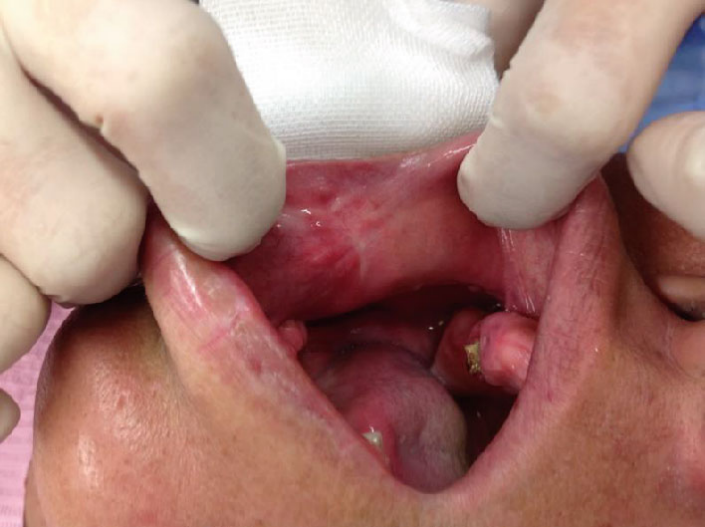
Postoperative intraoral. There is a scar in the mouth, but he has no trismus and no problem eating.

Lipomas are one of the most common types of benign, soft tissue tumors. Lipoma growth is slow, causing long periods of abnormality before diagnosis. The tumor was very large, pedunculated, and mobile; it was difficult to chew. Despite a masticatory and speech disorder, the patient described in this case report had lived with the lipoma for more than 15 years before diagnosis. The average period of noticing the presence of a lipoma to when they first seek treatment is 2 years [3]. Factors leading to lipoma development include (1) congenital internal factors, (2) endocrine imbalance, (3) relationship with nerves or peripheral nerves, (4) tuberculosis, and (5) continuous stimulation [4]. In this case, due to poor oral health and tooth loss, there was chronic irritation of the cheek by the remaining teeth, which may have contributed to lipoma formation. Lipomas often occur on the buccal mucosa, tongue, and floor of the mouth in 30- to 50-year-old patients. Interestingly, detailed case reports of lipoma have usually described lesion occurrence in men [3, 5]. Lipomas are often soft and occur submucosally or subcutaneously. However, in this case, the lipoma was pedunculated and about 40 mm in diameter, making this lesion the largest pedunculated lipoma to be observed in the oral cavity of a patient at our facility. Pedunculated lipomas in the oral cavity are rare, and such a large pedunculated lipoma was reported in only one previous case [6]. Lipomas may be classified as simple lipomas, fibrous lipomas, angiomyolipomas, myolipoma, myxoid lipoma, and callus lipoma [7]. They can originate from minor salivary glands. In this case, the patient presented with a simple lipoma, which was thought to originate from the minor salivary glands in the buccal mucosa. A biopsy was not performed before surgery, and the reason was that the course was very long, no nerve paralysis was observed, and MRI did not reveal infiltration of the surrounding area. And even if the tumor is too large and a part of it is resected for biopsy, it is difficult to diagnose it as malignant. Surgical excision is typically used to treat lipomas, and resection was performed immediately. Malignant changes are also rare [8]. A postoperative follow-up for one year indicated healing within normal limits, without any evidence of recurrence (Figure 5).

## 4. Conclusion

An extremely large lipoma (40 mm × 20 mm) was successfully excised from the buccal mucosa of a patient 15 years after initial presentation.

## Data Availability

The data that support the findings of this study are available from the corresponding author upon reasonable request.
